# The role of Self-Efficacy in practicing exclusive breastfeeding among female engineers

**DOI:** 10.1192/j.eurpsy.2026.11931

**Published:** 2026-07-31

**Authors:** A. Hrairi, N. Rmadi, F. Dhouib, A. Kchaou, O. Ben Ayed, E. Belkhir, M. Hajjaji, K. Jmal Hammami

**Affiliations:** 1 Occupational medicine department, Hedi Chaker university hospital; 2 Family medecine department, Faculty of medecine of Sfax; 3 Lactation consultant, Lactabelle society, Sfax, Tunisia

## Abstract

**Introduction:**

Exclusive breastfeeding (EBF) is recommended for the first six months of life as it provides essential energy and nutrients for infants. After returning to work, female engineers may face challenges in maintaining the EBF.

**Objectives:**

This study aimed to assess the influence of self-efficacy on breastfeeding success among female engineers in Tunisia.

**Methods:**

This was a cross-sectional study conducted in October 2024 in Tunisia, using an Arabic questionnaire created with Google forms and shared on social media. Eligible participants were female engineers under 45 years of age who had children aged 6 months to 5 years. In addition to collecting sociodemographic and professional data, three items from the Breastfeeding Self-Efficacy Scale short form (BSES-SF) was used to evaluate Self efficacy for practicing breastfeeding. The Self efficacy score was the sum of the three items.

**Results:**

A total of 51 female engineers were included, with a mean age of 32 ± 3.2 years (range : 25–39 years). Most participants (88.2%) worked in the private sector. The median number of working hours per week was 40 ± 3.8 (range : 25–48). Only 10.5% reported a positive breastfeeding experience, with a mean EBF duration of 4 ± 2.1 months. The BSES revaled that most participants kept wanting to breastfeed (mean=4.94 ; SD=0.238), managed the breastfeeding situation to their satisfaction (mean=4.06 ; SD=0.810), and coped successfully with breastfeeding like other challenging tasks (mean=3.51 ; SD=1.064). The Self efficacy score was significantly associated with the EBF (p=0.003).

**Conclusions:**

Mothers’ motivation to continue breastfeeding and avoid formula feeding is strongly influenced by their self-efficacy. Support for working women during maternity leave and upon returning to work is therefore recommended to promote sustained breastfeeding.

**Disclosure of Interest:**

None Declared

